# Downregulated miR-18a and miR-92a synergistically suppress non-small cell lung cancer via targeting *Sprouty 4*

**DOI:** 10.1080/21655979.2022.2066755

**Published:** 2022-04-29

**Authors:** Xinju Zhang, Xianyi Wang, Binshu Chai, Zong Wu, Xiaomin Liu, Heng Zou, Ziyi Hua, Zhongliang Ma, Weiwei Wang

**Affiliations:** a Lab for Noncoding RNA & Cancer, Shanghai University, Shanghai, China; b Experimental Center for Life Science, Shanghai University, Shanghai, China; c Department of Thoracic Surgery, The Third Affiliated Hospital of Kunming Medical University, Yunnan Cancer Hospital, Yunnan Cancer Center, Kunming, China

**Keywords:** miR-92a, miR-18a, SPRY4, NSCLC

## Abstract

As a novel noncoding RNA cluster, miR-17-92 cluster include six members: miR-17, miR-18a, miR-19a, miR-19b-1, miR-20a, and miR-92a. Dysregulation of miR-17-92 has been proved to be connected with the advancement of a series of human diseases, but the roles of miR-17-92 cluster in non-small cell lung cancer (NSCLC) have not been absolutely elaborated. Herein, we determined that miR-17-92 cluster were upregulated significantly in NSCLC tissues, and the cell proliferation, migration and cycle progression of NSCLC were also facilitated under the function of miR-17-92 cluster. *Sprouty 4* (*SPRY4*) was a direct target of miR-92a, and its overexpression restrained the exacerbation of NSCLC induced by miR-92a. Furthermore, the tumor xenograft assay showed that miR-92a facilitated tumor growth by inhibiting the expression of SPRY4 and mediating Epithelial-Mesenchymal Transition (EMT) *in vivo*. Finally, we looked into the synergistic effects of miR-92a and miR-18a on NSCLC, and found that antagomiR-18a treatment arrested the tumor growth rate of xenografted mice markedly.

## GRAPHICAL ABSTRACT

**Figure:**
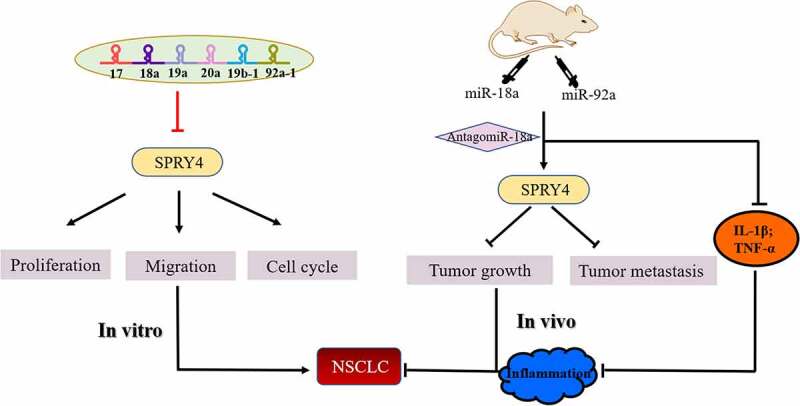

## Highlights

- MiR-17-92 cluster was upregulated in NSCLC tissues and cell lines.
- MiR-92a promoted the development of NSCLC in vitro and in vivo.
- AntagomiR-18a treatment limited the tumor growth of mice induced by miR-92a.
- SPRY4 is a common target of miR-17-92 cluster.

## Introduction

Lung cancer is one of the deadliest cancer in the world, and NSCLC occupies nearly 80% of all cases [1,2]. The 5-year overall survival for NSCLC patients is approximately 15% with a high recurrence rate [3,4]. Precision therapy is playing an increasingly important role in cancer treatment. Among which, miR-17-92 cluster as a oncogene cluster provides a feasible therapeutic target for the targeted therapy. MiR-17-92 cluster might be useful as a potential serum biomarker for the early detection of gastric cancer [5], and it also involved in the occurrence and development of bladder cancer [6]. Besides, miR-17-92 cluster could likely serve as a diagnostic marker in NSCLC [7]. These researches suggested that miR-17-92 cluster serving as a biomarker in some cancers could provide patients with precision therapy.

MicroRNAs (MiRNAs) are short non-coding RNAs with a length of 20–23 nt. Far-reaching effects on cancer progression and human carcinogenesis happened under the changes of miRNA expression profile [8]. MiRNA cluster is a class of miRNA that localize as clusters in the genome, whose transcripts are closely adjacent. As a result, they can regulate gene expression more widely [7,9]. MiR-17-92 cluster, which located on chromosome 13, is a highly conserved gene cluster and is the first oncogene cluster that has been discovered [10,11]. This cluster includes six miRNAs: miR-17, miR-18a, miR-19a, miR-19b-1, miR-20a, and miR-92a. Notably, miR-17 and miR-20a belong to miR-17 family because they only possess two different nucleotides. Similarly, miR-19a and miR-19b both belong to miR-19 family, with only one different nucleotide between them.

*SPRY4* takes an essential role in tumor suppression. Four members are included in SPRY family: SPRY1, SPRY2, SPRY3, and SPRY4. Studies have found that SPRY proteins serve as tumor suppressors in NSCLC via ERK pathway-dependent or ERK-independent mechanisms [12,13]. Furthermore, the members of SPRY family can also function to inhibit tumor growth by inhibiting the expression of EGFR or other growth factors [14]. Besides, it has been reported that SPRY4 is the target gene of the Wnt7A/Fzd9 pathway, which can inhibit the cell proliferation and invasion of NSCLC [15]. However, how the miR-17-92 cluster regulates the NSCLC growth through SPRY4 remains unclear.

Our previous research indicated that miR-17-92 cluster could be a diagnostic biomarker in NSCLC [7]. As a pivotal gene of miR-17-92 cluster, miR-18a has been discovered as an oncogene in lung cancer via targeting IRF2 [16]. However, how this cluster act on NSCLC and the specific mechanisms between them are rarely studied.

As previously mentioned, the miR-17-92 cluster can act as a diagnostic marker in a variety of tumors. So how does this cluster function in NSCLC? What is the specific mechanism between them? Does it play a corresponding role *in vivo*? These questions will be the focus of our research. In our study, we found that that miR-17-92 facilitated the tumorigenesis of NSCLC by downregulating the expression of SPRY4. Besides, antagomiR-18a treatment arrested the tumor growth rate of xenografted mice has also been confirmed *in vivo*. To conclude, our findings would provide a potential molecular therapeutic target for the treatment of NSCLC.

## Materials and methods

### Clinical tissues

NSCLC tissues were obtained from the Yunnan Cancer Hospital, Department of Thoracic Surgery (Yunnan, China), and were approved by the Ethics Committee of Yunnan Cancer Hospital (Approval No. KYCS2021206). Ninety-seven pairs (carcinoma and corresponding para-carcinoma) of NSCLC patients were recruited for this study. All the details of samples were listed in Supplementary Table S1.

### Cell culture

BEAS-2B, HBE, and HEK293T were obtained from the Cell Bank, China Academy of Sciences (Shanghai, China). A549, H1975, PC-9, and H1299 cells were purchased from the American Type Culture Collection (ATCC, Manassas, VA, USA).

A549, BEAS-2B, HEK293T, and PC-9 cells were cultured in DMEM (Gibco), supplemented with 10% fetal bovine serum (FBS, Gibco, Gaithersburg, MD, USA), and the antibiotic mixture of 100 μg/ml Streptomycin and 100 U/ml Penicillin (HyClone, Logan, UT, USA). H1975, H1299 cells were cultured in RPMI-1640 (Gibco), and the content of FBS and antibiotic as above. All cells were cultured in a 5% CO_2_ humidified environment at 37°C.

### RNA extraction, reverse transcription, and qRT-PCR

Total RNA of tissue and cell samples were extracted by Trizol (Sangon Biotech, Shanghai, China) following the manufacturer’s instruction, and was assessed as previously showed [17]. The PrimeScript^TM^ 1st Strand cDNA Synthesis Kit (TaKaRa, Dalian, China) and the PrimeScript® miRNA First-Strand cDNA Synthesis SuperMixQuantiMir cDNA Kit (Transgen Biotec, Beijing, China) was used to construct reverse transcription of mRNAs and miRNAs. The RNA levels of mRNA or miRNA were assessed by qRT-PCR using SYBR Green II (TaKaRa) and a CFX96^TM^ Real-time System (Bio-Rad). The 2^−ΔΔCt^ algorithm was used to calculate the relative expression of RNAs. The primer sequences were listed in Supplementary Table S2.

### Cell transfection

A549 and H1299 cells were transfected with miR-92a mimic/inhibitor, miR-18a mimic/inhibitor, SPRY4 siRNA (siSPRY4), or negative control (NC mimic/inhibitor and siNC) (RIBOBIO, Guangzhou, China) at concentrations of 50 nM, 50 nM, and 100 nM using Invitrogen™ Lipofectamine 2000 (Life Technologies, New York, USA) as previously showed [18]. After 48 h post-transfection, cells were performed for qRT-PCR, cell proliferation analysis, colony formation analysis, cell cycle analysis, and western blot.

### Cell proliferation assay

For CCK-8 assay, NSCLC cells were seeded into 96-well plates (Corning) at the density of 2,000 cells per well. After 24 h, 48 h, and 72 h of culture, 5% of CCK-8 was added into cells and incubated in the dark for 2 h. Then, the absorbance of CCK-8 treat cells at 450 nm was measured.

For colony formation assay, 300 or 600 cells were plated into 6-well plates (Corning) and continuously cultured for 12–14 days. After that, the colonies were fixed with methanol and stained with crystal violet (0.5% w/v).

### Wound healing assay

Cells were plated into 12 well plates (Corning) at the density of 2 × 10^5^ cells per well and until the density reaches 100%. Then, the cells were treated with sterile pipette tip to produce single scratch wounds and washed by PBS. After incubating with serum-free medium for 24 h or 48 h, the cell migration was assessed and photographed.

### Cell cycle analysis

2 × 10^5^ cells were seeded into 6-well plates and were collected and fixed in 75% ethanol after 24 h-48 h incubated. After centrifugation, the cells were washed once by PBS, and then dissolved in 250 mL RNaseA buffer (100 ng/mL; Sigma-Aldrich) at 37°C for 20 min. After that, they were stained with PI (50 ng/mL) in the dark for 15–20 min and filtered using a 200-mesh filter membrane. Finally, the results were determined with a MoFlo XDP flow cytometer (Beckman Coulter, USA) and the data were analyzed by Flow Jo software (Treestar Inc, USA).

### Dual luciferase reporter assay

Dual-luciferase reporter assay was assessed as previously showed [19]. The primer sequences used in the construction of pGL3-SPRY4-3ʹUTR and pGL3-SPRY4-3ʹmUTR plasmids were listed in Supplementary Table S2. HEK293T cells were cultured in 24-well plates until the cell density reached 60–80%, and transfected with 400 ng of pGL3-SPRY4-3ʹUTR or pGL3-SPRY4-3ʹmUTR, 20 ng of the renilla luciferase expression vector (pRL; Promega, Madi son, WI, USA) and 50 nM miR-92a mimic or NC mimic. After the 48 h of incubation, the luciferase activity was determined by an Orion II Microplate Illuminometer (Titertek-Berthold, South San Francisco, USA).

### Protein extraction and western blot analysis

Total proteins were obtained from the cells using RIPA lysis buffer (Leagene, Beijing, China) as previously showed [20] and quantified by a Protein BCA Assay Kit (Beyotime, Shanghai, China). After blocking with 5% powdered milk for at least 1.5 h to at room temperature, the membranes were incubated with primary antibodies against SPRY4 and GAPDH (1:1000, Cell Signaling Technology, Danvers, MA, USA) at 4°C overnight respectively. The PVDF membrane were washed with the Tris-buffered saline Tween 20 (TBST) for 3–4 times, and incubated with horseradish peroxidase (HRP)-conjugated secondary antibodies (1:10,000, Cell Signaling Technology, Danvers, MA, USA) for 1 h at room temperature. Subsequent visualization was detected with a chemiluminescent HRP substrate (Millipore Corporation, Billerica, USA) and imaged with an E-Gel Imager (Tanon, Shanghai, China).

### Subcutaneous tumor xenograft assay and metastatic assay

The 6 weeks old female null mice were purchased from the SLRC Laboratory Animal Center (Shanghai, China) and kept in a specific pathogen-free condition. The mice were injected with cells at a number of 5 × 10^6^ (resuspended in 100 μl DMEM medium) subcutaneously. The sizes of tumor tissues in mice were measured weekly and the volumes were calculated using the formula: volume = length × width^2^/2. To estimate the metastatic ability, mice were injected with 2.5 × 10^6^ cells (resuspended in 200 μl DMEM medium) via the tail vein, and after the experiment, the lung tissues were isolated to finish hematoxylin and eosin staining or immunohistochemistry [20]. All protocols used in our animal experiments were approved by the Ethics Committee of Yunnan Cancer Hospital (Approval No. KYCS2021206).

### Immunohistochemistry

The expression of Ki67, SPRY4, N-cadherin and E-cadherin in xenograft tumors were determined by immunohistochemistry. Primary tumor tissues were fixed with 10% formalin, embedded in paraffin, and cut into slices with 4 μm thickness. The following procedure was performed as previous description [21], and the slices were treated with primary antibodies against Ki67 (1:200; Servicebio, Wuhan, China), E-cadherin, N-cadherin, and SPRY4 (1:500–1:200, Cell Signaling Technology), followed by goat anti-rabbit HRP-conjugated antibodies applying. Finally, the samples were treated with a 3,3’-diaminobenzidine reaction solution, and imaged by a digitalized microscope camera.

### Statistical analysis

Experiments were performed independently at least thrice. Mean ± S.E.M represented the data and their differences, and the t-test was used to analyze the significance of the data. Furthermore, the results were statistically significant when the p < 0.05. Graph Pad Prism 7 software was used to analyze the results.

## Results

In this study, more attention was paid to investigating the clinical significance and potential molecular mechanism of miR-17-92 in NSCLC. We verified the upregulation of miR-17-92 in NSCLC by database and qRT-PCR assay. We demonstrated the pro-cancer effect of miR-17-92 *in vitro* and *in vivo*. The relationship between SPRY4 and miR-17-92 cluster was proved by the dual-luciferase reporter assay. Besides, we found that ectopic expression of SPRY4 rescued the function of miR-92a in NSCLC. AntagomiR-18a restrained the driving effects of miR-92a on the development of NSCLC and decreased the immune level of mice induced by miR-92a and miR-18a overexpression. In summary, we speculated that miR-17-92 might be involved in the tumorigenesis of NSCLC through targeting SPRY4.

### MiR-92a facilitated the tumorigenesis of NSCLC

Our previous study has proved that miR-17-92 cluster can be served as a blood-based marker for cancer detection in NSCLC [7]. Moreover, according to the analysis of dbDEMC 2.0, the members of miR-17-92 cluster are all upregulated in lung cancer (Supplementary Figure S1(a)). This conclusion was also been verified in NSCLC tissues (Supplementary Figure S1(b)). It is worth noting that miR-92a had a highest expression level among the members of miR-17-92 cluster. Therefore, in this paper, we focused on miR-92a as our key point.

**Figure 1.**
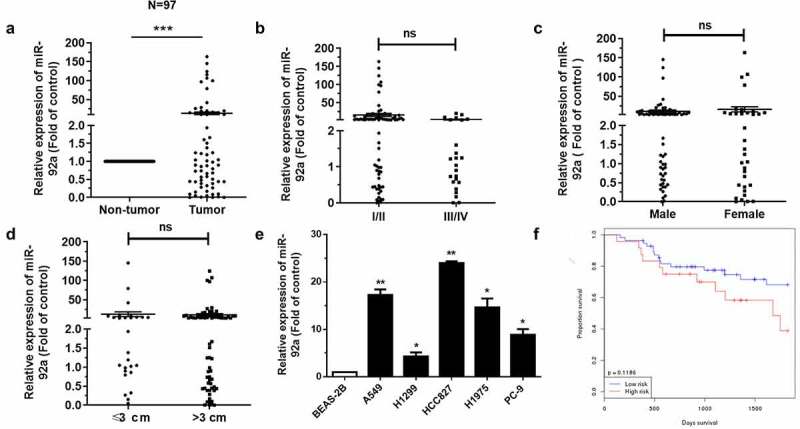
MiR-92a facilitated the tumorigenesis of NSCLC.

MiR-92a, as a classical oncogene, could promote the epithelial-mesenchymal transition (EMT) in NSCLC [22]. However, what role does miR-92a play in NSCLC and which key gene it regulates remain unknown. In addition, how the miR-92a functions on the progression of NSCLC *in vivo* remain poorly studied. Accordingly, we detected expression levels of miR-92a in patients through qRT-PCR first of all. The expression of miR-92a had a significant upregulation in tumor tissues, compared with non-tumor lung tissues (Figure 1(a)). However, the expression levels of miR-92a had no relation with the pathological stage, gender, and tumor size (Figure 1(b–d)). Studies indicated that miR-92a was much higher in NSCLC cell lines, compared with BEAS-2B cell (Figure 1(e)). We also found that the patients of longer survival have lower miR-92a expression (Figure 1(f)). Collectively, these findings indicated that the upregulated miR-92a increased carcinogenesis of NSCLC.

(a) MiR-92a was upregulated in NSCLC tissues. (b-d) qRT-PCR analysis of miR-92a expression related to pathological stage (b), tumor gender (c) and size (d). (e) qRT-PCR analysis of miR-92a expression in BEAS-2B and NSCLC cell lines A549, H1299, HCC827, H1975, and PC-9. (f) Relationship between miR-92a expression and patient survival. U6 was used as an internal control. Error bars represent the mean ±S.E.M. *P < 0.05, **P < 0.01, ***P < 0.001.

### MiR-92a promoted cell proliferation, migration, and accelerated cell cycle in NSCLC

**Figure 2.**
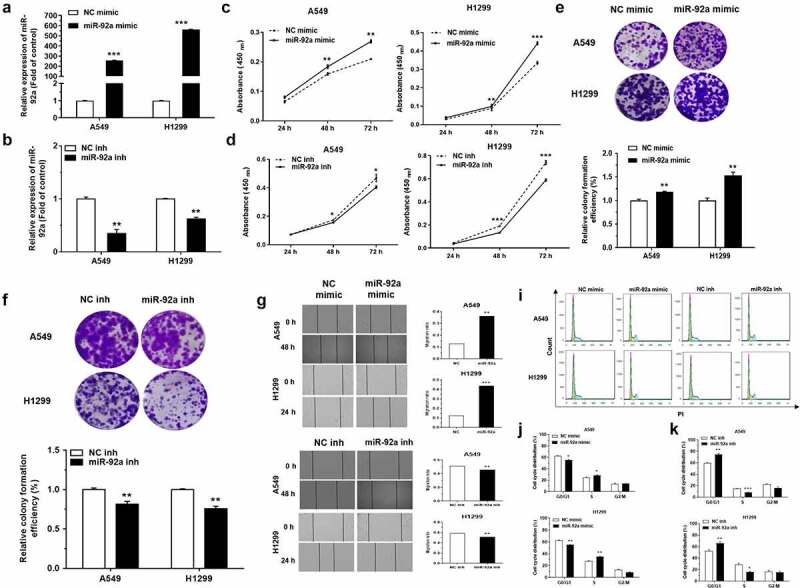
MiR-92a promoted cell proliferation, migration and accelerated cell cycle in NSCLC.

The administration of miR-92a mimic was used to increase the expression level of miR-92a. According to the results of qRT-PCR, the transfection of miR-92a mimic indeed upregulated the expression of miR-92a in A549 and H1299 cells (Figure 2(a)). Meanwhile, the expression of miR-92a was blocked using specific inhibitor, and examined by qRT-PCR (Figure 2(b)). The effects of miR-92a on NSCLC development was verified by CCK-8 and colony formation assays. MiR-92a overexpression induced an apparent increase in cell growth during 24 h-72 h assessed by CCK-8 assay and colony formation assay (Figure 2(c,e)). In the same way, the cell proliferative abilities were also arrested enormously under the inhibition of miR-92a in A549 and H1299 cells (Figure 2(d,f)). Moreover, the changes of cell migration after miR-92a overexpression and downregulation were characterized by the wound healing assay (Figure 2(g–h)). These results signified that miR-92a enhanced the cell proliferation and migration ability of NSCLC *in vitro*.

(a-b) qRT-PCR detected the expression of miR-92a in H1299 and A549 cells, with NC/miR-92a mimic transfection (A) or NC/miR-92a inhibitor transfection (B). U6 was used as an internal control. (c-d) CCK-8 assay detected the cell proliferation ability of H1299 and A549 cells transfected with NC/miR-92a mimic (C), and NC/miR-92a inhibitor (D). (e-f) Representative images and quantification of colony formation ability in H1299 and A549 cells transfected with NC/miR-92a mimic (E) and transfected with NC/miR-92a inhibitor (F). (g-h) Representative images and quantification of cell migration rate in H1299 and A549 cells with NC/miR-92a mimic transfection (G), and NC/miR-92a inhibitor transfection (H). (i-k) Representative images (I) and quantification of the cell cycle distributions in H1299 and A549 cells, transfected with NC/miR-92a mimic (J), and NC/miR-92a inhibitor (K). Error bars represent the mean ± S.E.M. *P < 0.05, **P < 0.01, ***P < 0.001.

A549 and H1299 cells transfected with miR-92a mimic and miR-92a inhibitor were cultured in serum-free medium for 6 hours, arresting the cells for the same period, and then returning to normal culture to restore cell cycle progression. The results of flow cytometry showed that miR-92a overexpression could facilitate the progression of cell cycle evidently, but miR-92a inhibition exhibited the opposite effects (Figure 2(i–k)).

### SPRY4 was a direct target of miR-92a

**Figure 3.**
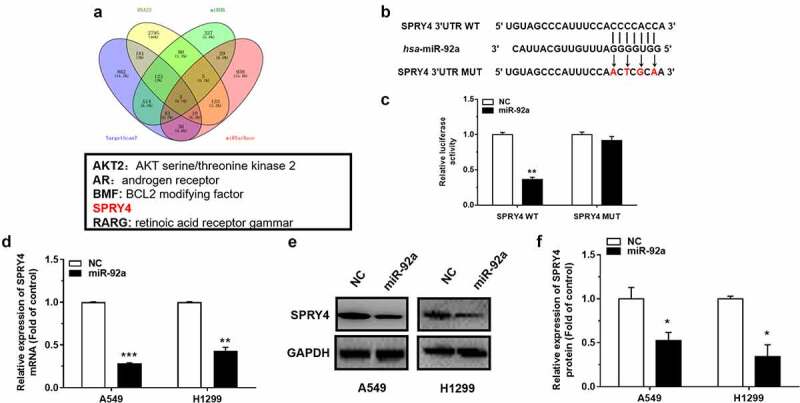
SPRY4 is a direct target of miR-92a.

To investigate the potential target of miR-92a, we first predicted the possible target genes of miR-92a using Targetscan (http://www.targetscan.org/vert_72/), RNA22 (https://cm.jefferson.edu/rna22/), miRDB (http://mirdb.org/), and miRTarBase (https://maayanlab.cloud/Harmonizome/resource/MiRTarBase). Then, we analyzed the results obtained from these five databases, and we found that SPRY4 was predicted in every database. Our previous study has proved that SPRY4 could serve as a direct target of miR-411 and play a tumor suppressor role in NSCLC [23]. However, how miR-92a targets SPRY4 to function in NSCLC has not been studied, therefore we identified SPRY4 as a potential target for miR-92a (Figure 3(a)). The correlation between miR-92a and SPRY4 was verified by the dual-luciferase reporter assay. The site-directed mutagenesis for 3’-UTR of SPRY4 was designed to destroy the binding between miR-92a and SPRY4 (Figure 3(b)). The results showed that the relative luciferase activity decreased more than 50% in the HEK-293 T cell transfected with SPRY4-WT-3’-UTR compared with SPRY4-Mut-3’-UTR transfection (Figure 3(c)), which indicated that miR-92a could target SPRY4 via the sequence specificity. In further validation, decreased mRNA and protein levels of SPRY4 upon miR-92a mimic transfection in NSCLC cell lines were proved by both qRT-PCR and western blot assay (Figure 3(d–f)). The above results support that SPRY4 is a direct target of miR-92a.

(a) Five candidate genes of miR-92a are screened by targetscan, RNA22, miRDB, and miRTarBase. (b) The predicted wild-type (WT) and corresponding mutant (Mut) binding sites of SPRY4 3’-UTR with miR-92a. (c) The relative luciferase activity in HEK293T cells, co-transfected with pGL3-SPRY4-WT-3’-UTR/Mut-3’-UTR, pRL and NC/miR-92a mimic. (d) qRT-PCR detected the expression of SPRY4 mRNA in H1299 and A549 cells with miR-92a overexpression. (e-f) Western blot (E) and quantification (F) of SPRY4 in H1299 and A549 cells with miR-92a overexpression. Error bars represent the mean ± S.E.M. *P < 0.05, **P < 0.01, ***P < 0.001.

### SPRY4 functioned to suppress NSCLC

**Figure 4.**
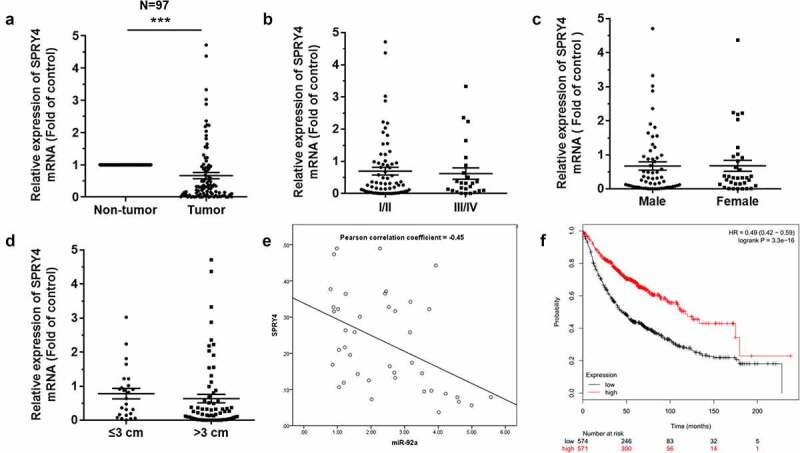
SPRY4 was downregulated in NSCLC.

Among 97 pairs of tissues, SPRY4 was downregulated significantly, as compared to that of corresponding para-carcinoma tissues (Figure 4(a)). However, the expression of SPRY4 has little relationship with the pathological stage (Figure 4(b)), gender (Figure 4(c)), or tumor size (Figure 4(d)). Besides, the analysis of the Pearson Correlation Coefficient revealed that there is a negative correlation between miR-92a and SPRY4 (Figure 4(e)). The Kaplan–Meier survival analysis of NSCLC patients showed a positive correlation between SPRY4 expression and patient survival. Among the 1145 cases, NSCLC patients with high survival rates exhibit the higher expression of SPRY4 (Figure 4(f)).

(a) qRT-PCR detected the expression of SPRY4 in 97 pairs of NSCLC tissue samples. (b-d) qRT-PCR analysis of SPRY4 expression related to pathological stage, sex, and tumor size in NSCLC tissues. (e) Pearson correlation coefficient detected the negative correlation between miR-92a and SPRY4. (f) The overall survival of 1145 lung cancer patients from Kaplan-Meier Plotter online database. Error bars represent the mean ± S.E.M. *P < 0.05, **P < 0.01, ***P < 0.001.

**Figure 5.**
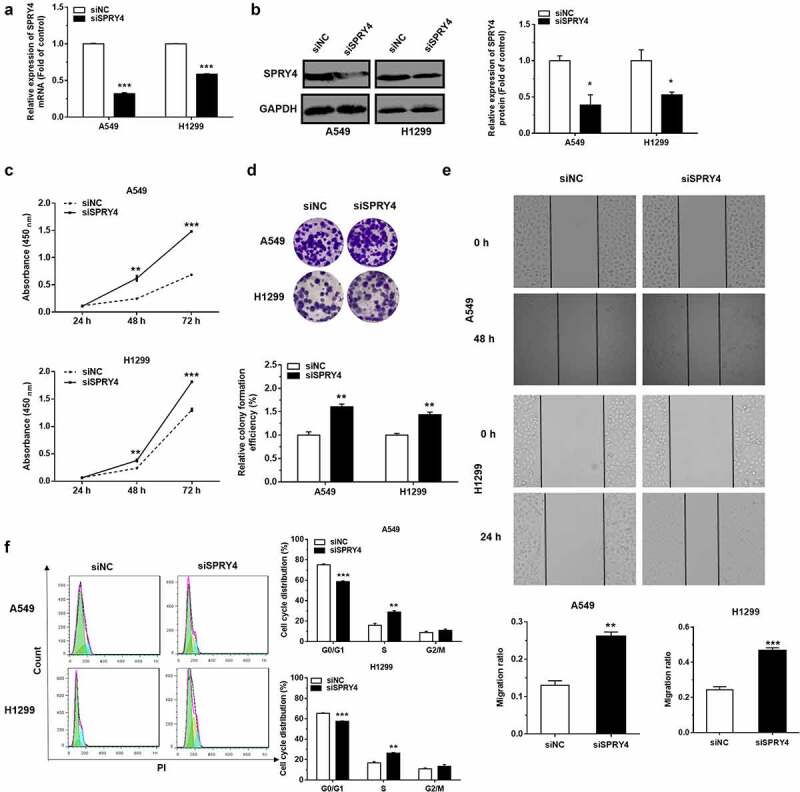
SPRY4 functioned to suppress NSCLC.

To investigate the biological effects of miR-92a on NSCLC, the small-interfering RNA (siRNA) was used to downregulate the expression of SPRY4, and then the changes in cell phenotypes were detected in NSCLC cells [24,25,25]. The effects of siRNA transfection on SPRY4 were confirmed by mRNA and protein detection (Figure 5(a,b)). The cell proliferation and migration abilities of H1299 and A549 transfected with siSPRY4 were promoted significantly (Figure 5(c–e)). Moreover, after 48 h of SPRY4 knockdown, the results of flow cytometric analysis showed that siSPRY4 facilitated the conversion of cell cycle from G0/G1 phase to S phase significantly in NSCLC cells (Figure 5(f)). Together our data showed that SPRY4 knockdown exhibited similar effects as miR-92a overexpression, which highlighted that the exacerbated effects of miR-92a on NSCLC development were partially mediated by targeting SPRY4.

(a-b) The mRNA (a) and protein (b) levels of SPRY4 in H1299 and A549 cells transfected with siNC or siSPRY4. 18S rRNA and GAPDH were used as internal control for qRT-PCR and Western blot, respectively. (c-e) Representative images and quantification of cell proliferation (c), colony formation (d) and migration ability (e) in H1299 and A549 cells with siSPRY4 transfection. (f) Representative images and quantification of the cell cycle distributions in H1299 and A549 cells with siSPRY4 transfection. Error bars represent the mean ± S.E.M. *P < 0.05, **P < 0.01, ***P < 0.001.

### Ectoptic expression of SPRY4 rescued the function of miR-92a in NSCLC

**Figure 6.**
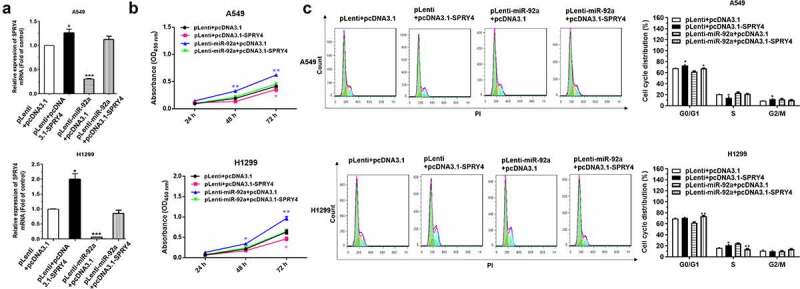
SPRY4 could rescue the effects of miR-92a on NSCLC.

In order to prove that the function change caused by miR-92a overexpression is due to the SPRY4 downregulation, we constructed the miR-92a stable overexpression NSCLC cell models by pLenti-miR-92a or pLenti lentivirus infection. The positive cells for green fluorescence were screened after Flow cytometry sorting (Supplementary Figure S2(a)). The miR-92a expression levels of pLenti-miR-92a cells were upregulated significantly, with approximately 20- and 65-fold increase, respectively. (Supplementary Figure S2(b)). CCK8 assay was performed to validate the cancer-promoting effects of pLenti-miR-92a cells on NSCLC proliferation (Supplementary Figure S2(c)). Then, the pcDNA3.1-SPRY4 was transfected into pLenti-miR-92a A549 and H1299 cell lines to overexpress SPRY4. Subsequently, the expression level of SPRY4 were increased enormously after pcDNA3.1-SPRY4 transfection in pLenti-miR-92a NSCLC cell lines, which indicated that pcDNA3.1-SPRY4 rescued the declined expression of SPRY4 caused by miR-92a overexpression successfully (Figure 6(a)).

(a) The expression level of SPRY4 in miR-92a-stably overexpressing (pLenti-miR-92a) and negative control (pLenti) A549 and H1299 cells following transfection with SPRY4 expression vector (pcDNA3.1-SPRY4) and negative control (pcDNA3.1). (b) The proliferation rate of A549 and H1299 cells (pLenti-miR-92a/pLenti) following transfection with pcDNA3.1-SPRY4 and pcDNA3.1, as measured by Cell Counting Kit-8 assay. (c) The cell cycle distributions of A549 and H1299 (pLenti-miR-92a/pLenti) cells following transfection with pcDNA3.1-SPRY4 and pcDNA3.1 were analyzed by flow cytometry. Error bars represent the mean ± S.E.M.*P < 0.05, **P < 0.01, ***P < 0.001.

The results of CCK-8 assay and Flow cytometry analysis revealed that SPRY4 overexpression obviously reversed the promotion function of miR-92a overexpression on cell proliferation and cell cycle in NSCLC (Figure 6(b,c)). Collectively, we demonstrated that SPRY4 upregulation could rescue the functions of miR-92a overexpression in NSCLC.

### *miR-92a boosted the progression of NSCLC* in vivo

**Figure 7.**
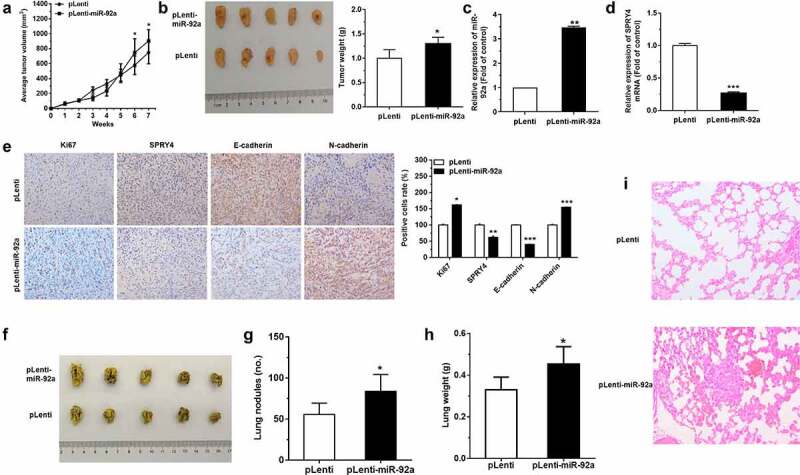
miR-92a boosted the progression of NSCLC *in vivo.*

In order to evaluate the tumor-promoting effects of miR-92a *in vivo*, we injected nude mice with pLenti-miR-92a or pLenti A549 cells at a number of 5 × 10^6^ subcutaneously and measured tumor volume weekly. pLenti-miR-92a A549 cell group exhibited the rapid increase in tumor growth and weight at 7 weeks post-implantation (Figure 7(a,b)). Moreover, in pLenti-miR-92a group, we observed a marked miR-92a overexpression, with a reverse trend in SPRY4 expression (Figure 7(c,d)). Furthermore, the immunohistochemistry results indicated that the cell proliferation marker, Ki67 and epithelial mesenchymal transition (EMT) marker, N-cadherin were increased, with E-cadherin and SPRY4 decrease in miR-92a overexpression group (Figure 7(e)).

(a) The volume of tumor growth was measured weekly after injection. (b) Representative images and weights of xenograft tumors with pLenti or pLenti-miR-92a A549 cells injection. (c-d) qRT-PCR detected the expression of miR-92a (C) and SPRY4 (D) in subcutaneous xenograft tumors. (e) Immunohistochemistry staining of Ki67, SPRY4, E-cadherin, and N-cadherin in xenograft tumor tissues (original magnification × 100). (f) The lungs of mice with metastasis nodes are displayed. (g-h) The numbers of nodules (g) and weight of lungs (h) in mice with pLenti-miR-92a and pLenti A549 cells injection. (i) Pathological sections of lung metastases in the nude mice (original magnification ×100). Error bars represent the mean ± S.E.M. *P < 0.05, **P < 0.01, ***P < 0.001.

MiR-92a overexpression A549 cells were also injected intravenously into the tail vein of nude mice to detect the function of miR-92a on tumor metastasis *in vivo*. More massive and confluent metastatic nodes were observed in pLenti-92a group after 7 weeks of tail vein injection (Figure 7(f–g)). The weight of lungs also increased a lot after miR-92a overexpression (Figure 7(h)). Moreover, the results of HE staining in the bilateral lungs showed more necrotic or destructive regions in miR-92a overexpression group, whereas the control group exhibited fewer and smaller tumor nests (Figure 7(i)). Collectively, these findings demonstrated that miR-92a can facilitate tumor growth and metastasis through the repression of SPRY4 and the induction of EMT *in vivo*.

### AntagomiR-18a restrained the driving effects of miR-92a on the development of NSCLC

Unlike miR-17 and miR-19 families, which have two members, respectively, miR-18 and miR-92 families only have one corresponding member, and this indicates that they may have more powerful biological function due to their sequence specificity. Consequently, based on our previous research that miR-18a and miR-92a are proved as oncogenes in NSCLC, respectively, we assumed that the combined effects of the miR-18a and miR-92a could play more significant roles in the development of NSCLC. Therefore, we conducted the follow-up study.

**Figure 8.**
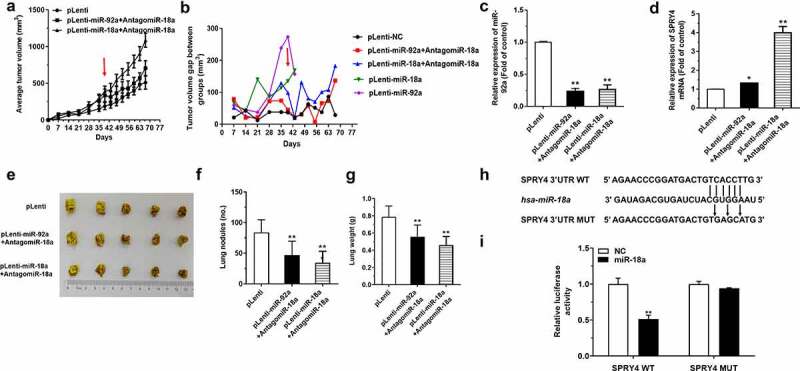
AntagomiR-18a restrained the driving effects of miR-92a on the development of NSCLC.

Firstly, nude mice were divided into three groups randomly and injected with pLenti-miR-92a, pLenti-miR-18a, and pLenti A549 cells subcutaneously. After the subcutaneous tumor reached a certain volume about 5 weeks, the tumor-bearing mice were treated with antagomiR-18a around the formed subcutaneous tumor on the 39th day, and the corresponding tumor size was measured every 4 days (Figure 8(a)). According to the results, although the subcutaneous tumor is still increasing, the growth rate is much lower than before (Figure 8(b)). In addition, the expression level of miR-92a was downregulated significantly in the tumor tissues of the pLenti-miR-92a+AntagomiR-18a and pLenti-miR-18a+AntagomiR-18a groups (Figure 8(c)), accompanied by the upregulated expression of SPRY4 (Figure 8(d)). Furthermore, the metastatic nodes of the miR-18a and miR-92a groups treated with antagomiR-18a become more massive and confluent compared with those in the control group. Then, the lung weights in the mice of miR-18a and miR-92a groups with antagomir-18atreatment were constricted a lot (Figure 8(e–g)). Because the tumor-promoting effects of miR-18a were accompanied with a decrease in SPRY4 expression, we explored the mechanism by which miR-18a cooperated with miR-92a through SPRY4. We found that there was also a potentially targeted binding site of miR-18a with SPRY4 (Figure 8(h)), and this binding effect was further verified by the dual-luciferase reporter assay. Compared with the SPRY4-Mut-3’-UTR transfection, the relative luciferase activity was decreased a lot in the cells transfected with SPRY4-WT-3’-UTR (Figure 8(i)). We therefore confirmed that it is the binding of miR-18a to SPRY4 that leads to the synergistic promotion effects of miR-18a and miR-92a on the tumor growth of mice. In other words, the administration of antagomiR-18a reduced the decrease in SPRY4 levels induced by the direct binding of miR-18a to SPRY4.

(a) Tumor volume of subcutaneous xenograft tumors (pLenti, pLenti-miR-92a, and pLenti-miR-18a) treated with antagomiR-18a on 39th day were measured weekly, and the growth curves were generated. (b) Changes of mice volume in subcutaneous xenograft tumors following treated with antagomiR-18a. (c-d) qRT-PCR detected the expressions of miR-92a (C) and SPRY4 mRNA (D) in xenograft tumors following antagomiR-18a treatment. (e) The lungs of mice with metastasis nodes are displayed. (f-g) The numbers of lung nodules (f) and the weight of lungs (g) in mice with antagomiR-18a treatment. (h) The binding sites of SPRY4 3’-UTR with miR-18a are predicted. (i) The relative luciferase activity in HEK293T cells, co-transfected with pGL3-SPRY4-WT-3’-UTR/Mut-3’-UTR, pRL and NC/miR-18a mimic. Error bars represent the mean ± S.E.M. *P < 0.05, **P < 0.01, ***P < 0.001.

### AntagomiR-18a decreased the immune level of mice induced by miR-92a and miR-18a overexpression

As for the tumor microenvironment, the rapid growth of cells and inadequate supply of oxygen and blood nutrients will induce the release of some proinflammatory mediators, such as IL-1 and HMGB1 [26]. Additionally, miR-17-92 cluster has been proved to be critical in lung cancer microenvironment, such as the regulation of T cell response, the lymphoma development, autoimmunity, and so on [8,27,28]. Therefore, we want to know what roles immune response plays in the synergistic effect of miR-18a and miR-92a on tumor growth. We took the spleen tissues from the mice with antagomiR-18a treatment and determined the expression changes of several inflammatory factors in it. From the results of qRT-PCR assay, we can see that antagomiR-18a could obviously downregulate the expression of miR-18a in the spleen but have no obvious effects on miR-92a expression (Supplementary Figure S3(a, b)). Then, we detected the expression of several proinflammatory mediators, including IL-1β, TNF-α, and IL-6. We found that except for IL-6, the levels of IL-1β and TNF-α decreased significantly after antagomiR-18a applying (Supplementary Figure S3(c–e)). These results suggested that immune response might be involved in the antago-miR-18a treatment of tumor growth in mice, which would provide a new and feasible solution for cancer treatment.

## Discussion

MiR-92a has been found participated in different types of diseases. Li et al. investigated the carcinogenic roles of miR-92a in colorectal cancer cells. They found that miR-92a inhibition attenuated sphere formation capacity, decreased expressions of stemness-related proteins, and inhibited the proliferation of cancer stem-like cells [29]. MiR-92a was also found as a molecular sponge for circRbms1 to modulate the expression of apoptosis-related genes [30]. However, as a member of the miR-17-92 cluster, whether miR-92a interacts with other members of the cluster to jointly regulate the development of diseases is still poorly studied. In our study, we found that miR-92a can coordinate with miR-18a to promote the development of NSCLC, and the administration of antagomiR-18a can effectively inhibit the synergistic effects of the two.

As a way of accurate treatment, miRNA regulation has been widely used in tumor therapy. The oncogenic role of the miR-17-92 cluster has been demonstrated in different types of tumors [31], and they exert effects in tumorigenesis via the regulation of various target genes, respectively [32]. In this study, the upregulated miR-92a was detected in NSCLC tissues and cell lines. Overexpression of miR-92a promoted tumorigenesis *in vitro*, which mainly manifested in the control of cell proliferation, migration, and cell cycle. Moreover, miR-92a could promote tumor growth and metastasis through E-cadherin downregulation, Ki67, and N-cadherin upregulation. SPRY4 as a classic tumor suppressor has been confirmed in many researches [15,23,33]. In our study, it is found that the expression of SPRY4 were negatively correlated to the expression of miR-92a *in vitro*. This regulation is dependent on the binding between miR-92a and the 3’-UTR of SPRY4, which would recruit the RNA-induced silencing complex (RISC) to degrade the mRNA of SPRY4, thereby inhibiting the expression of SPRY4 mRNA, and further affecting its protein level. Moreover, one previous study in our lab has shown that miR-18a can significantly promote NSCLC tumor progression by directly targeting IRF2 [16]. Combined with the current results, we hypothesized that miR-92a and miR-18a will co-regulate tumorigenesis. In order to validate, our hypothesis, a preliminary exploration of the correlation between miR-92a and miR-18a was carried *in vivo*. Our study demonstrates that antagomiR-18a treatment could inhibit the progression of NSCLC facilitated by miR-92a overexpression. As for the synergistic effects between miR-92a and miR-18a, we found that both miR-18a and miR-92a can target SPRY4 through the dual-luciferase reporter assay, so it proves that miR-18a and miR-92a may play tumor-promoting roles by targeting SPRY4 commonly. SPRY4, as a tumor suppressor gene, was found that it has a good anti-tumor effect in vivo and in vitro and ectopic expression of SPRY4 rescued the function of miR-92a in NSCLC. Besides, in vivo experiments showed that antagomiR-18a could inhibit the tumor-promoting effects of miR-92a, which also exhibit a interaction between them.

Combined with the current discoveries on the roles of miR-17-92 cluster in different cancers, it has been paid more and more attention in NSCLC. Our research not only opens up a new chapter for the exploration of the relationship between miRNAs and tumors, but also suggests that there may be more complex relationships between miRNAs and tumorigenesis, which requires us to continually explore. On the whole, the synergistic relationship between miR-92a and miR-18a and the therapeutic effects of antagomiR-18a may provide new targets for the clinical diagnosis and treatment of lung cancer.

## Conclusion

In our study, we demonstrated that miR-17-92 cluster can significantly promote the development of NSCLC by inhibiting *SPRY4*, and there is a synergistic effect between the members of miR-17-92 cluster *in vitro* and *in vivo*. In conclusion, our findings may provide a new therapeutic target for lung cancer.

## Supplementary Material

Supplemental Material

## Data Availability

The data that support the findings of this study are available from the corresponding author upon reasonable request.zlma@shu.edu.cn
