# Supplementary material for: Downregulated miR-18a and miR-92a synergistically suppress non-small cell lung cancer via targeting Sprouty 4
Source: Bioengineered. 2022 Apr 29;13(4):11281–95. doi: 10.1080/21655979.2022.2066755 (PMC9208480; doi:10.1080/21655979.2022.2066755)
Supplement: Supplemental Material [file KBIE_A_2066755_SM8987.docx]

**Supplementary Figure 1. MiR-17-92 cluster expression profile in NSCLC**

**
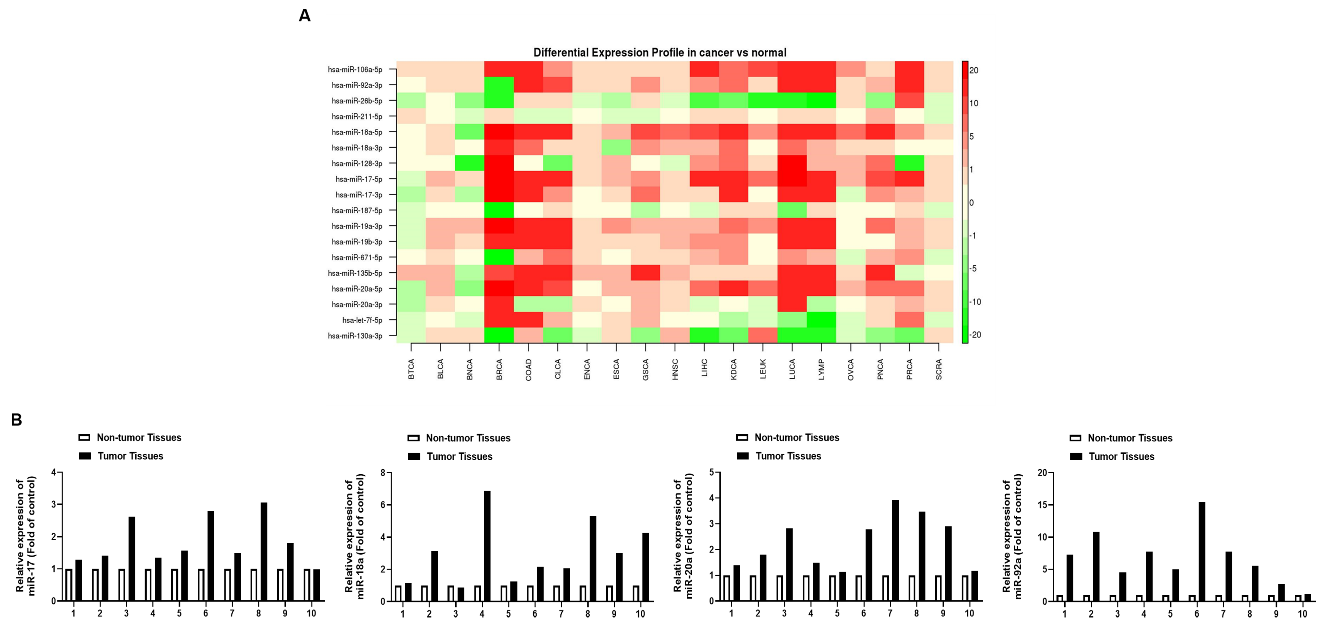
**

**(A)** Expression profiles of miR-17-92 cluster from two groups of different cancer types. **(B)** qRT-PCR analysis of miR-17-92 cluster expression in cancer and corresponding para-carcinoma tissues from NSCLC patients. U6 was used as an internal control. Error bars represent the mean ± S.E.M.*P<0.05, **P<0.01, ***P<0.001.

**Supplementary Figure 2.** **Construction of miR-92a stable overexpression cell lines**

**
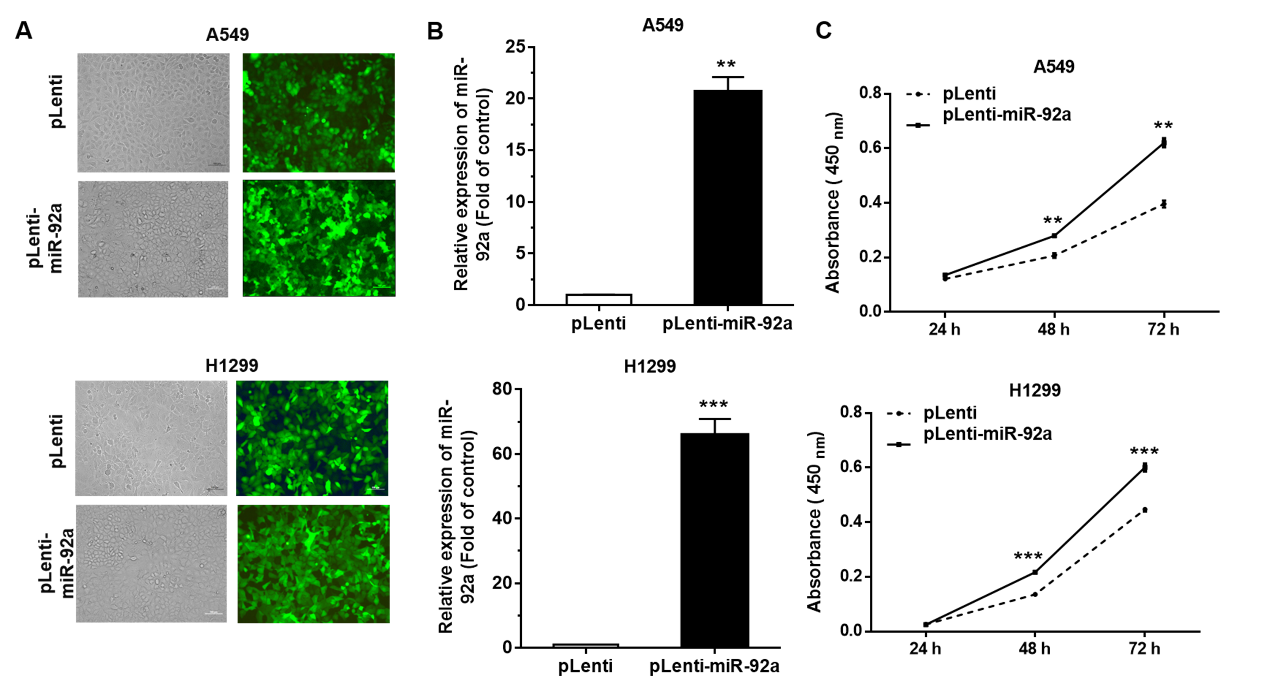
**

1. The positive pLenti-miR-92a A548 cell and H1299 cell were shown by imaging with inverted fluorescence microscope. **(B)** qRT-PCR analysis of miR-92a expression in A549 and H1299 cells, stably transfected with pLenti/pLenti-miR-92a by lentivirus infection. **(C)** Cell proliferation ability of A549 and H1299 cells stably transfected with pLenti/pLenti-miR-92a. Error bars represent the mean ± S.E.M.*P<0.05, **P<0.01, ***P<0.001.

**Supplementary Figure 3. Downregulation miR-18a decreased the immune level of mice.**

**
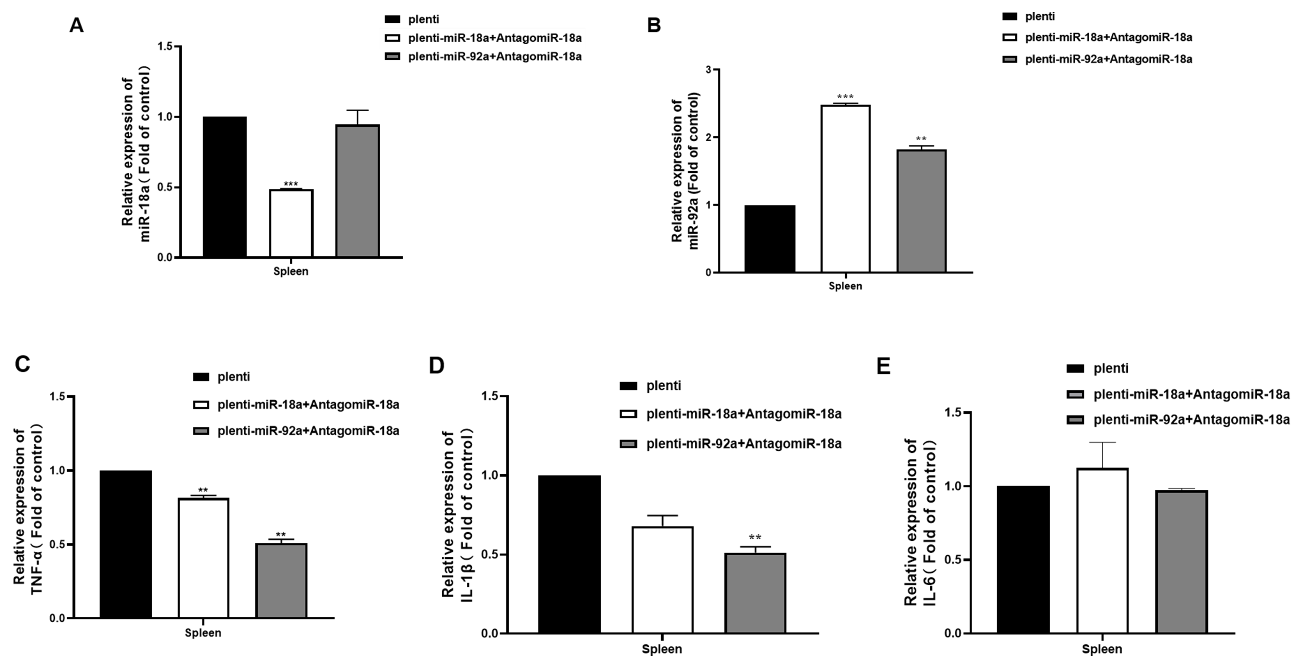
**

**(A-B)** qRT-PCR analysis of miR-18a (A) and miR-92a expression (B) in the spleen of nude mice injected with pLenti, pLenti-miR-92a and pLenti-miR-18a A549cells after treated with antaogomiR-18a. **(C-E)** qRT-PCR analysis of TNF-α (C), IL-1β (D) and IL-6 expression (E) in the spleen of nude mice injected with pLenti, pLenti-miR-92a and pLenti-miR-18a A549cells after treated with antaogomiR-18a. Error bars represent the mean ± S.E.M.*P<0.05, **P<0.01, ***P<0.001.

**Supplementary Table S1**

The clinical-pathological features of 97 cases NSCLC patients

| NO. | Gender | Histologic Type | Lymphatic Invasion | | pTNM |
| --- | --- | --- | --- | --- | --- |
| 1 | M | Adenocarcinoma | Absent | T2a | |
| 2 | F | Adenocarcinoma | Absent | T2a | |
| 3 | F | Adenocarcinoma | Absent | T2a | |
| 4 | F | \| Adenocarcinoma \| \| --- \| | Absent | T2a | |
| 5 | M | \| Squamous cell \| \| --- \| \| carcinoma \| | Absent | T2a | |
| 6 | M | Adenocarcinoma | Absent | T4 | |
| 7 | M | Adenocarcinoma | Present | T3 | |
| 8 | M | Adenocarcinoma | Absent | T1a | |
| 9 | F | Adenocarcinoma | Absent | T2a | |
| 10 | M | Adenocarcinoma | Absent | T4 | |
| 11 | M | \| Squamous cell \| \| --- \| \| carcinoma \| | Present | T4 | |
| 12 | F | \| Squamous cell \| \| --- \| \| carcinoma \| | Present | T3 | |
| 13 | F | \| Squamous cell \| \| --- \| \| carcinoma \| | Present | T2N1M0 | |
| 14 | M | \| Squamous cell \| \| --- \| \| carcinoma \| | Present | T3N2M0 | |
| 15 | M | Adenocarcinoma | Absent | T1aN0M0 | |
| 16 | M | Adenocarcinoma | Absent | T1bN0M0 | |
| 17 | M | \| Squamous cell \| \| --- \| \| carcinoma \| | Present | T2aN0M0 | |
| 18 | M | Adenocarcinoma | Present | T2aN0M0 | |
| 19 | M | \| Squamous cell \| \| --- \| \| carcinoma \| | Present | T1bN0M0 | |
| 20 | M | Adenocarcinoma | Absent | T2aN0M0 | |
| 21 | M | Adenocarcinoma | Present | T2aN0M0 | |
| 22 | M | Adenocarcinoma | Absent | T2a | |
| 23 | F | Adenocarcinoma | Absent | T1bN0M0 | |
| 24 | M | Adenocarcinoma | Absent | T2aN0M0 | |
| 25 | M | \| Squamous cell \| \| --- \| \| carcinoma \| | Present | T1b | |
| 26 | F | \| Adenocarcinoma \| \| --- \| | Absent | T2a | |
| 27 | M | Adenocarcinoma | Absent | T1bN0M0 | |
| 28 | M | Adenocarcinoma | Absent | T2bN0M0 | |
| 29 | F | Adenocarcinoma | Present | T1bN2M0 | |
| 30 | M | \| Squamous cell \| \| --- \| \| carcinoma \| | Absent |  | |
| NO. | Gender | Histologic Type | Lymphatic Invasion | pTNM | |
| 31 | M | Adenocarcinoma | Absent | T2aN0M0 | |
| 32 | M | Adenocarcinoma | Absent | T1aN0M0 | |
| 33 | F | Adenocarcinoma | Absent | T1bN0M0 | |
| 34 | M | Adenocarcinoma | Absent | T2aN0M0 | |
| 35 | M | Adenocarcinoma | Absent | T2aN0M1a | |
| 36 | F | Adenocarcinoma | Absent | T3N0M0 | |
| 37 | M | Adenocarcinoma | Absent | T1bN0M0 | |
| 38 | M | Adenocarcinoma | Absent | T2aN0M0 | |
| 39 | F | Adenocarcinoma | Absent | T2aN0M0 | |
| 40 | F | Adenocarcinoma | Present | T1bN0M0 | |
| 41 | F | Adenocarcinoma | Present |  | |
| 42 | M | Multiple cell carcinoma | Present | T1bN0M0 | |
| 43 | M | Adenocarcinoma | Absent | T2aN2M0 | |
| 44 | F | Adenocarcinoma | Absent | T2aN0M0 | |
| 45 | F | Adenocarcinoma | Absent | T2bN1M0 | |
| 46 | M | Adenocarcinoma | Absent | T2aN0M0 | |
| 47 | M | Adenocarcinoma | Present | T2aN0M0 | |
| 48 | F | Adenocarcinoma | Present | T2aN0M0 | |
| 49 | M | Adenocarcinoma | Absent | T2aN2M0 | |
| 50 | M | Adenocarcinoma | Absent | T2aN0M0 | |
| 51 | M | \| Squamous cell \| \| --- \| \| carcinoma \| | Present | T2aN0M0 | |
| 52 | M | Adenocarcinoma | Present | T2aN1M0 | |
| 53 | M | Adenocarcinoma | Absent | T2aN0M0 | |
| 54 | M | Adenocarcinoma | Present | T2aN2M0 | |
| 55 | M | Adenocarcinoma | Present | T2aN0M0 | |
| 56 | M | \| Squamous cell \| \| --- \| \| carcinoma \| | Present | T2aN0M0 | |
| 57 | F | Adenocarcinoma | Absent | T2aN0M0 | |
| 58 | F | Adenocarcinoma | Absent | T2aN0M0 | |
| 59 | F | \| Squamous cell \| \| --- \| \| carcinoma \| | Present | T2bN1M0 | |
| 60 | F | Adenocarcinoma | Absent | T2aN2M0 | |
| 61 | F | Adenocarcinoma | Absent | P-T1bN0M0 | |
| 62 |  | Adenocarcinoma | Absent | P-T2aN1M0 | |
| 63 | M | Adenocarcinoma | Absent | P-T2bN2M0 | |
| 64 | F | Adenocarcinoma | Absent | P-T4N3M0 | |
| 65 | F | Adenocarcinoma | Absent | P-T2aN3M0 | |
| 66 | F | Adenocarcinoma | Present | P-T1bN0M0 | |
| 67 | M | Adenocarcinoma | Present | P-T3N0M0 | |
| 68 | F | Adenocarcinoma | Absent | P-T1bN0M0 | |
| 69 | F | Adenocarcinoma | Absent | P-T2aN1M0 | |
| NO. | Gender | Histologic Type | Lymphatic Invasion | pTNM | |
| 70 | M | Adenocarcinoma | Present | P-T1bN0M0 | |
| 71 | M | Adenocarcinoma | Present | P-T2bN1M0 | |
| 72 | F | Adenocarcinoma | Absent | P-T2aN0M0 | |
| 73 | M | Adenocarcinoma | Absent | P-T2bN1M0 | |
| 74 | M | \| Squamous cell \| \| --- \| \| carcinoma \| | Absent | P-T2aN0M0 | |
| 75 | M | Adenocarcinoma | Absent | P-T2aN0M0 | |
| 76 | F | Adenocarcinoma | Absent | P-T3N0M0 | |
| 77 | M | Adenocarcinoma | Present | P-T2aN0M0 | |
| 78 | F | Adenocarcinoma | Present | T2aN1M0 | |
| 79 | F | Adenocarcinoma | Absent | T2aN0M0 | |
| 80 | F | Adenocarcinoma | Absent | T2aN2M0 | |
| 81 | F |  | Present | T2aN0M0 | |
| 82 | M | \| Squamous cell \| \| --- \| \| carcinoma \| | Present | T2aN0M0 | |
| 83 | F | Adenocarcinoma | Absent | T2aN0M0 | |
| 84 | F | Adenocarcinoma | Absent | T2aN0M0 | |
| 85 | F | Adenocarcinoma | Absent | T2bN1M0 | |
| 86 | M | Adenocarcinoma | Absent | T2aN1M0 | |
| 87 | M | Adenocarcinoma | Absent | T2aN0M0 | |
| 88 | F | Adenocarcinoma | Present | T2aN2M0 | |
| 89 | F | Adenocarcinoma | Present | T2aN0M0 | |
| 90 | F | Adenocarcinoma | Absent | T2aN0M0 | |
| 91 | F | Adenocarcinoma | Absent | T2aN0M0 | |
| 92 | F |  | Present | T2aN0M0 | |
| 93 | M |  | Present | T2bN1M0 | |
| 94 | M | Adenocarcinoma | Absent | T2a | |
| 95 | F | Adenocarcinoma | Absent | T2a | |
| 96 | M | Adenocarcinoma | Absent | T2aN0M0 | |
| 97 | M | Adenocarcinoma | Present | T2aN1M0 | |

**Supplementary Table S2**

The sequence of the primers used in this study

| Primer | Sequence（5'-3'） |
| --- | --- |
| 18S RNA(F) | AGGAATTCCCAGTAAGTGCG |
| 18S RNA(R) | GCCTCACTAAACCATCCAA |
| U6 snRNA(F) | CTCGCTTCGGCAGCACA |
| U6 snRNA(R) | AACGCTTCACGAATTTGCGT |
| SPRY4-3'-UTR(F) | GCTCTAGACTTGCCTTGCTTTCTCTT |
| SPRY4-3'-UTR(R) | CGGAATTCGCAGTCCCGTGTATATTTAAC |
| SPRY4-3'-mUTR(F) | TAGCCCATTTCCAACTCGCAACAAAGTTGCCACAGCTT |
| SPRY4-3'-mUTR(R) | AACCTGTGGCAACTTTGTTGCGAGTTGGAAATGGGCTA |
| SPRY4 qRT-PCR(F)  SPRY4 qRT-PCR(R)  pcDNA3.1(-)-SPRY4(F)  pcDNA3.1(-)-SPRY4(R)  siSPRY4 | CGGAAAATACAGAGACCACC  GGACCCTGAAAAAAAGCC  GCTCTAGAATGGAGCCCCCGATCCCACAGA  CGGAATTCTCAGAAAGGCTTGTCGGGCCTGC  CAGCACATCCTCTGACCAA  CTGCAGAATGAGGACGAT  TGTGGAGAATGACTACATA |
| miR-92a qRT-PCR  miR-18a qRT-PCR | GGGTGGGGATTTGTTGCATTAC  TAAGGTGCATCTAGTGCAGATAG |
| pri-miR-92a(F)  pri-miR-92a(R)  pri-miR-18a(F)  pri-miR-18a(R) | GGATCCACACCTTCATGCGTATCTCC  CTCGAGCCATCTAGCTTACTGTAGCG  CGGGATCCGTGAAGGCACTTGTAGCATT  CCCTCGAGCAAAACTAACAGAGGACTGC |
